# Reference Genes for Circadian Profiling of Core Clock Genes in the Blood of Obstructive Sleep Apnea Patients

**DOI:** 10.3390/biom16071013

**Published:** 2026-07-10

**Authors:** Katarina Nahtigal, Ana Halužan Vasle, Tinkara Kreft, Cene Skubic, Miha Mraz, Miha Moškon, Leja Dolenc Grošelj, Damjana Rozman

**Affiliations:** 1 Centre for Functional Genomics and Bio-Chips, Institute of Biochemistry and Molecular Genetics, Faculty of Medicine, University of Ljubljana, 1000 Ljubljana, Slovenia; katarina.nahtigal@mf.uni-lj.si (K.N.); tinkara.kreft@mf.uni-lj.si (T.K.); cene.skubic@mf.uni-lj.si (C.S.); leja.dolenc-groselj@mf.uni-lj.si (L.D.G.); 2 Computational Biology Group, Faculty of Computer and Information Science, University of Ljubljana, 1000 Ljubljana, Sloveniamiha.mraz@fri.uni-lj.si (M.M.); miha.moskon@fri.uni-lj.si (M.M.)

**Keywords:** circadian rhythms, obstructive sleep apnea, reference genes, qRT-PCR normalization, gene expression stability, core clock genes

## Abstract

Circadian rhythm disruptions are increasingly recognized in disorders such as obstructive sleep apnea (OSA), yet analysis of 24 h gene expression patterns remains challenging due to the lack of reliable reference genes for normalization. Even commonly used housekeeping genes may exhibit circadian oscillations, which can confound rhythmic gene expression analyses and hinder biomarker identification. To address this limitation, we evaluated the gene expression stability of 11 commonly used housekeeping genes in blood collected every 6 h over 24 h period from 40 adults with varying OSA severity and controls. Stability ranking by analytical tools RefFinder and EndoGeneAnalyzer identified *ACTB* (β-actin) and *RPL13A* (ribosomal protein L13a) as the most consistent reference genes, with minimal intra- and inter-individual variability across sampling times and disease groups. Their suitability was assessed by personalized cosinor analysis of core clock genes (*BMAL1*, *PER2*, *CRY1*), demonstrating that appropriate normalization enables detection of circadian oscillations in clinical samples. Using the optimal normalization, CosinorPy analysis of the core clock genes revealed significant circadian oscillations of at least one clock gene in the studied participants. These findings establish *ACTB* and *RPL13A* as robust reference genes for blood-based circadian studies of OSA and provide an important methodological framework for future circadian biomarker research.

## 1. Introduction

Despite advances in transcriptomic technologies, quantitative reverse transcription polymerase chain reaction (qRT-PCR) remains the gold standard for low-throughput gene expression analysis. Accurate qRT-PCR measurements depend on RNA quality, reverse transcription and PCR efficiency, and particularly on the selection of appropriate reference genes for normalization [1,2]. Because reference gene suitability is context-dependent, circadian studies require genes with stable 24 h expression profiles. However, this is challenging since the circadian clock regulates a wide range of physiological processes, including the expression of commonly used reference genes themselves.

Circadian rhythm is an adaptation of living organisms to the natural cycle of environmental light and darkness, with an intrinsic period of approximately 24 h [3,4]. Most living organisms exhibit observable daily rhythms that arise from internal, self-sustaining circadian clocks. In mammals, the master regulator of this timing system resides in the suprachiasmatic nucleus (SCN), located in the anterior hypothalamus. Notably, individual SCN neurons have been shown to maintain their own circadian oscillations, indicating that the underlying clock mechanism operates at the cellular level [5]. While the SCN functions as the master pacemaker, near-identical molecular clock machinery exists in peripheral tissues, including the cells of the hematopoietic lineage, where it oscillates autonomously rather than relying directly on neuronal input [6,7]. Synchronization between the central and peripheral clocks is achieved indirectly, through behavioral rest–activity and feeding cycles, and directly, via a humoral and neural relay. The SCN signals through the hypothalamic–pituitary–adrenal axis drive rhythmic release of glucocorticoids and catecholamines, while local sympathetic innervation provides an additional, tissue-specific synchronizing cue through cyclic noradrenaline release [7]. Because blood cells are not innervated by the SCN directly, their clock gene oscillations are entrained largely through the circulating humoral signals together with the sleep–wake cycle. These cues act through shared neuroendocrine pathways, including circadian and sleep-dependent regulation of cortisol, sympathetic activity, and growth hormone secretion [8]. As a consequence, peripheral clock gene rhythms in leukocytes are not simply a passive readout of the SCN. Their phase and amplitude can diverge or become uncoupled from the central pacemaker rhythms [6,9]. This partial autonomy means that disturbances in peripheral entrainment pathways, such as the intermittent hypoxia and sleep fragmentation seen in obstructive sleep apnea (OSA), may alter clock gene expression in blood cells even when the central clock remains unaffected. This provides a strong rationale for directly measuring circadian clock gene expression in the buffy coat of OSA patients rather than relying solely on central clock function. On a molecular level, the circadian rhythm is controlled by the transcriptional and translational feedback loops of core clock components. The central clock involves transcription factors (e.g., BMAL1 and CLOCK) activating clock-controlled genes (*PER* and *CRY* gene families), which in turn inhibit the activators and produce self-sustained oscillations [3].

This biological clock is intrinsic to almost all life forms and has gained increasing interest, particularly due to its impact on physiology. Circadian rhythms influence a variety of physiological processes, from metabolism, regulation of body temperature, endocrine secretion and even labor onset [10]. Understanding the genetic mechanisms that drive circadian rhythms is crucial, as disruptions in these rhythms are linked to health conditions [11], such as metabolic disorders (e.g., metabolic dysfunction-associated steatotic liver disease [12,13,14], cardiovascular diseases (e.g., heart failure) [15], and sleep disturbances (e.g., OSA) [16].

We aim to identify reliable circadian biomarkers in blood cells of patients with OSA, a disorder characterized by repeated breathing interruptions during sleep, fragmented sleep, intermittent hypoxia, and multiple health complications [3,17] that overlap with other diseases associated with circadian rhythm disruption [4]. Accumulating evidence indicates that OSA disrupts the molecular circadian clock, and it has been hypothesized that OSA severity correlates with distinct patterns of clock gene expression in peripheral blood cells [3]. Therefore, a prerequisite for this investigation is the identification of stable reference genes from human blood cells for normalizing the circadian gene expression [18,19]. Since most validated datasets are still rodent-based, human circadian normalization studies remain scarce, which limits accurate and reproducible circadian gene expression studies in humans [18,19,20,21,22,23,24]. Although human studies on circadian reference gene validation are gradually emerging, they have largely been conducted in tissues and not in the blood or under conditions that do not reflect the OSA milieu. Hadadi et al. [18] validated reference genes for circadian expression in human mammary epithelial cells, and White et al. [25] performed a comparable analysis in human adipose tissue, neither of which translates directly to blood-derived samples. The most relevant blood-based study by Ackermann et al. [26] examined clock gene rhythms in peripheral blood of healthy volunteers subjected to acute total sleep deprivation, providing a useful methodological reference but one that does not account for the intermittent hypoxia, chronic sleep fragmentation, and immune cell composition shifts that characterize OSA. Ledderose et al. [19] validated reference genes specifically in human T cells and neutrophils, yet without a circadian or disease-specific framework. Collectively, no existing study has validated reference genes under the combined challenges of 24 h temporal sampling in humans, variable disease severity, and intermittent hypoxia, underscoring the need for context-specific validation in this patient population.

Therefore, the primary goal of this study was to determine which housekeeping genes exhibit the most stable expression profiles across different time points and different OSA severities. We present a 24 h analysis of candidate reference gene expression of RNA, isolated from the buffy coat (the leukocyte–thrombocyte layer) of blood from patients with varying OSA severity. Our results identify two reference genes that are most suitable for normalizing circadian expression of core clock genes, paving the foundation for future circadian biomarker discovery in OSA.

## 2. Materials and Methods

### 2.1. Subjects and Blood Sample Collection

Forty participants (16 women and 24 men, aged 18–65 years) with clinically suspected OSA underwent overnight respiratory polygraphy (Alice NightOne; Philips Respironics, Amsterdam, the Netherlands, EU) in an outpatient clinical setting at the Clinical Institute of Clinical Neurophysiology, University Medical Centre Ljubljana, Slovenia. The study was approved by the Commission for Medical Ethics of the Republic of Slovenia (approval No. 0120-65/2023/3) and conducted in accordance with its guidelines. All participants were of the same ethnic background and provided written informed consent in the presence of the responsible physician. As part of the study inclusion process, participants completed the Morningness–Eveningness Questionnaire to determine their chronotype, and those with an extreme morning or evening chronotype were excluded from the study. Participants were classified by apnea–hypopnea index (AHI) into four groups: control (AHI < 5), mild (AHI 5–14.9), moderate (AHI 15–29.9), and severe (AHI ≥ 30) OSA, each comprising 10 individuals.

Before the overnight respiratory polygraphy, the participants were given written and oral instructions on timing and composition of meals, physical activity limitation, and directions about sleep hygiene. Participants were asked to eat a low-fat dinner before 18:00 at home; not to eat before morning sampling (at 7:00); and abstain from drinking coffee and alcohol throughout the study protocol. The polygraph started recording at 22:00 and ended at 6:00 the next day. During that time, patients were instructed not to use electronic devices, turn off the light, and to go to sleep as soon as possible. Any interaction between medical staff and patients during the night was performed using dim light.

During the study, peripheral venous blood (3 mL per draw, EDTA anticoagulant) was collected via an indwelling catheter at five time points over 24 h: T0, 13:00; T1, 19:00; T2, 01:00; T3, 07:00; T4, 13:00 (following day). Within 30 min of each draw, samples were centrifuged (2000× *g*, 15 min, 4 °C), plasma was removed, and 300 µL of buffy coat was collected for RNA isolation. Additionally, total and differential white blood cell counts were measured on the morning samples collected at 7:00 (T3).

### 2.2. RNA Isolation, cDNA Preparation, and qRT-PCR

Total RNA was extracted from the buffy coat using TRI Reagent LS (Sigma-Aldrich GmbH, Schnelldorf, Germany, EU); the detailed protocol is provided in the Appendix A. RNA quantity and purity were assessed by spectrophotometry (NanoDrop 1000; Applied Biosystems, Thermo Fisher Scientific, Waltham, MA, USA). Complementary DNA was synthesized with the QuantiTect Reverse Transcription Kit (Qiagen GmbH, Hildren, Germany, EU). qRT-PCR was performed using PowerTrack SYBR Green Master Mix (Applied Biosystems, Thermo Fisher Scientific, Waltham, MA, USA) on a QuantStudio 5 instrument (Applied Biosystems, Thermo Fisher Scientific, Waltham, MA, USA), following the manufacturer’s recommended cycling conditions. All reactions were run in triplicate with no-template controls, and amplification specificity was confirmed by melt-curve analysis (single peak per primer set).

### 2.3. Primer Design

Eleven candidate reference genes were selected based on published literature [1,15,19,20,22,23,27,28,29,30,31,32] and previous work from our group [21]: *GAPDH*, *SDHA*, *PPIB*, *ACTB*, *CDK4*, *HPRT1*, *RPL13A*, *TUBB2A*, *PPIA*, *UBC*, and *TBP*. Additional primer sets were tested for selected genes (denoted ‘#2’) to evaluate alternative splice variants or amplicon targets (*SDHA* #2, *PPIB* #2, *CDK4* #2). Primers were designed with NCBI Primer-BLAST, version 2.5.0 (Table S1). Amplification efficiency for each primer pair was determined by standard curve analysis from a 6-point, 2-fold serial dilution series of pooled cDNA (dilution factors 1×, 2×, 4×, 8×, 16×, and 32×). For each dilution, the crossing threshold was plotted against the natural logarithm of the relative cDNA amount, and the slope of the resulting linear regression was used to calculate efficiency (E) according to E = 10^(–1/slope) [33]. Only primer pairs with efficiency between 90 and 110% were retained, and specificity was confirmed by gel electrophoresis. Primer sequences, amplicon lengths, standard curve regression parameters and amplification efficiencies are listed in Table S2. Raw quantification cycle (Cq) data were managed with QuantStudio Design and Analysis Software version 1.6.1.

### 2.4. Reference Gene Stability Analysis

Reference gene stability was assessed using two complementary tools. RefFinder [34] integrates four established algorithms: geNorm [2,35], NormFinder [36], BestKeeper [33], and the comparative ΔCt method [37]. It generates a geometric mean-based comprehensive ranking (lower score = higher stability). EndoGeneAnalyzer [38] complements RefFinder by accommodating complex, multi-factor designs. It handles multiple grouping variables (e.g., disease group, time point), removes outliers, and computes NormFinder-based stability metrics within each subgroup. This enabled evaluation not only across the full dataset but also within specific conditions (OSA severity, 24 h intra-individual variation, and time of day). Reference gene selection followed a stepwise elimination approach guided by results from both tools (Section 3.1).

### 2.5. Cosinor Analysis of Core Gene Rhythmicity

To validate the selected reference genes, the rhythmic expression of core clock genes *BMAL1*, *CRY1*, and *PER2* was examined by cosinor rhythmometry, a statistical method that fits a cosine function to time-series data and tests for a 24 h oscillation. The fitted model yields three biologically meaningful parameters: the mesor (rhythm-adjusted mean expression level), the amplitude (half the peak-to-trough difference, indicating oscillation magnitude), and the acrophase (time at which expression peaks). Cq values were normalized using an efficiency-corrected relative quantity method. For each gene, the relative quantity was calculated as E^(−Cq), where E denotes the amplification efficiency factor (E = 1 + efficiency/100). To obtain a sample-scaled relative expression, each value was divided by the maximum observed expression across all samples for the respective gene. The normalization factor (NF) was computed as the geometric mean of the scaled expression values of *ACTB* and *RPL13A*, further adjusted relative to the global average geometric mean across all samples, following the multi-reference gene normalization principle described by Vandesompele et al. [35]. Normalized expression (NE) was thus defined as NE = (E_target_^(−Cq_target_)/max[E_target_^(−Cq)])/NF. Cosine curve fitting was performed with CosinorPy [39]. Both population-level (group-averaged) and personalized (individual-level) cosinor models were constructed to capture group trends and inter-individual variation, respectively. For personalized cosinor analysis, rhythmicity was assessed separately for each participant and target gene. To account for multiple comparisons arising from testing multiple genes across multiple participants, *p*-values were adjusted using the false discovery rate (FDR) procedure performed globally. Adjusted *p*-values < 0.05 were considered statistically significant and reported as q-values.

## 3. Results

### 3.1. Reference Gene Selection

**Figure 1:**
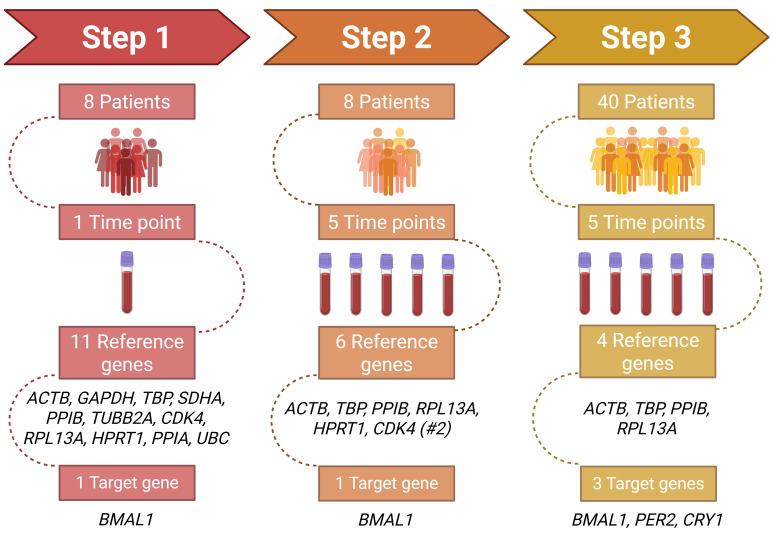
Schematic overview of the three-step reference gene selection process in this study. The study was conducted in three sequential steps. In each step, we increased the scope of analysis (either the number of participants, the number of blood sampling time points, and/or the number of target genes) while correspondingly narrowing down the list of candidate reference genes. At each step, the elimination of reference gene candidates was based on the stability results obtained with algorithms implemented in online tools RefFinder and EndoGeneAnalyzer. Step 1 involved 8 participants sampled at a single time-point to evaluate the expression stability of 11 candidate reference genes, using *BMAL1* as a representative target gene. In step 2, we used the same 8 patients and included samples from all 5 time-points to assess circadian expression patterns and refine the selection to the 6 most stable reference genes. In step 3, the final analysis was expanded on a cohort of 40 patients, sampled at 5 time-points, using the 4 most stable reference genes (*ACTB*, *TBP*, *PPIB*, *RPL13A*) from previous steps for normalization. Expression of three core clock genes (*BMAL1*, *PER2*, and *CRY1*) was evaluated in this final phase. “#2” denotes an alternative primer sequence targeting a different transcript of the same gene.

Forty participants were allocated to three OSA severity and control groups (10 per group) based on AHI. Baseline demographic, chronotype, anthropometric, and hematological characteristics of the study groups are presented in Table S3. From all the presented parameters, only body mass index (BMI) showed statistical significance between groups (*p* < 0.05). Reference gene selection proceeded through three sequential steps (Figure 1), each expanding the analytical scope while narrowing the candidate pool.

**Step 1**: Single time-point screening (*n* = 8). Eleven candidate reference genes (*ACTB*, *GAPDH*, *TBP*, *SDHA*, *PPIB*, *TUBB2A*, *CDK4*, *RPL13A*, *HPRT1*, *PPIA*, *UBC*), three alternative primer sets (*SDHA* #2, *PPIB* #2, *CDK4* #2), and *BMAL1* (representative target gene) were evaluated in eight participants (2 per OSA group) at T0 (13:00). Because all samples were collected at the same circadian time, data were grouped by OSA severity and analyzed primarily with RefFinder. Six genes (*GAPDH*, *SDHA*, *TUBB2A*, *CDK4*, *PPIA*, *UBC*) and the *SDHA* #2 and *PPIB* #2 variants were eliminated due to high variability (Figure S1, Table S4, Figure S2, Table S5). Although the small sample size (*n* = 2 per OSA severity group) limits the statistical power of this screening step, Step 1 was intentionally designed as a rapid preliminary filter to exclude genes with clearly high variability before proceeding to the more resource-intensive multi-timepoint analysis in Steps 2 and 3.

**Step 2**: Circadian time-series assessment (*n* = 8, five time points). The six best-performing genes (*ACTB*, *TBP*, *PPIB*, *CDK4* #2, *RPL13A*, *HPRT1*) were re-evaluated across all five time points in the same eight participants using EndoGeneAnalyzer with three grouping factors: individual patient, OSA severity, and time of day. The results (Figure S3, Table S6) showed that some genes had consistently low stability in all scenarios. In particular, *HPRT1* and *CDK4 #2* emerged as less stable (higher variability) across conditions. RefFinder (Figure S4, Table S7) also placed these two genes in mid-to-low stability range. Therefore, we eliminated *HPRT1* and *CDK4 #2* at this stage.

**Figure 2:**
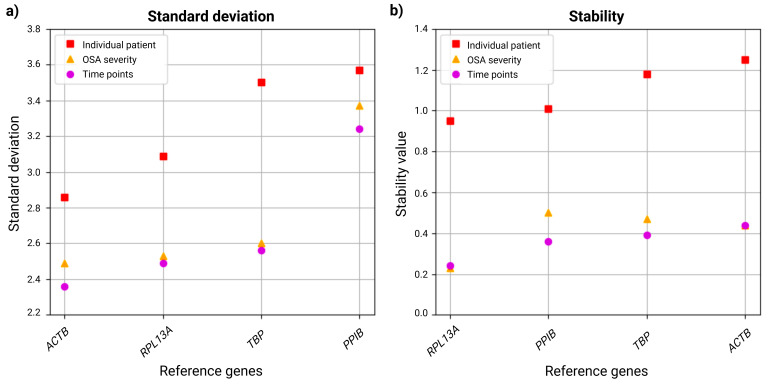
EndoGeneAnalyzer results of the four candidate reference genes in Step 3. Standard deviation (**a**) and stability (**b**) of the four best-performing reference genes (*ACTB*, *RPL13A*, *TBP*, *PPIB*) are shown based on three grouping criteria: individual patient (red squares), OSA severity (orange triangles), and time points (purple circles). Lower standard deviation and stability values indicate more consistent gene expression. *ACTB* consistently exhibited the lowest variability (**a**) within all tested conditions, while *RPL13A* showed the lowest stability values (**b**) across all grouping conditions.

**Step 3**: Full cohort validation (*n* = 40, five time points). The four remaining genes (*ACTB*, *TBP*, *PPIB*, *RPL13A*) were assessed across all 40 participants at five time points. Three clock genes (*BMAL1*, *CRY1*, *PER2*) were measured concurrently for downstream rhythmicity analysis. EndoGeneAnalyzer results (Figure 2) showed that *ACTB* had the lowest standard deviation across all three grouping conditions (Figure 2a), while *RPL13A* achieved the lowest stability values (Figure 2b; lower = more stable), followed closely by *ACTB*. *TBP* ranked lowest on both metrics. Detailed values are in Table S8.

**Figure 3:**
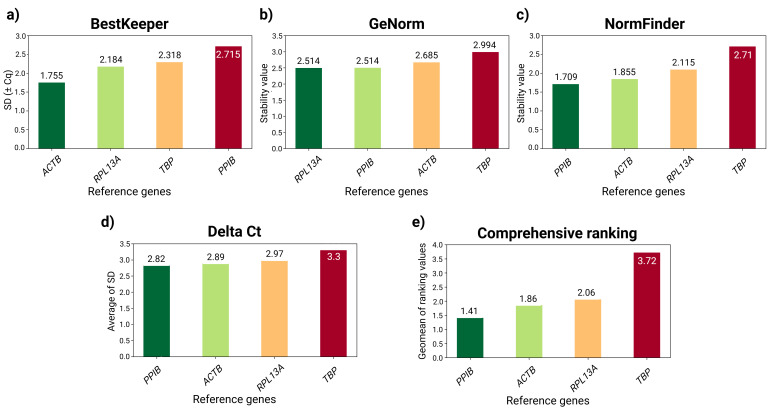
RefFinder analysis of reference gene stability in Step 3. The figure shows results from four algorithms (BestKeeper, geNorm, NormFinder, and the Delta Ct method) with additional comprehensive ranking. Each graph (**a**–**e**) represents one analysis method, plotting the stability or variability measures for *ACTB*, *RPL13A*, *PPIB*, and *TBP*. In all cases, lower values (left side) correspond to greater expression stability. *ACTB* was identified as the top performer by BestKeeper (**a**), *RPL13A* and *PPIB* ranked highest by geNorm (**b**), additionally, *PPIB* showed the best stability by NormFinder (**c**), lowest average of SD by ∆Ct method (**d**) and overall best geometric mean of ranking values by comprehensive ranking (**e**). *TBP* consistently showed the lowest stability in four algorithms (**b**–**e**).

RefFinder results (Figure 3, Table S9) were consistent: *ACTB* ranked first by BestKeeper and second by NormFinder, the ΔCt method, and the comprehensive score. *RPL13A* ranked first by geNorm, together with *PPIB*, which scored best by NormFinder, ΔCt, and comprehensive ranking. *TBP* was the least stable gene by all algorithms, except BestKeeper.

Combining evidence from both tools across all grouping conditions, *ACTB* and *RPL13A* were selected as the optimal reference genes. Both showed low intra- and inter-group variability and stable expression across the circadian cycle and were reliably detected in every sample on the first attempt. *PPIB* was excluded despite favorable algorithm scores because its Cq values were undetectable in a subset of 24 samples (all four groups and all time points) even after three repeated measurements, a practical limitation invisible to computational analysis alone. *TBP* was excluded due to poor stability across four out of five algorithms.

### 3.2. Assessment of Core Clock Gene Rhythmicity

**Figure 4:**
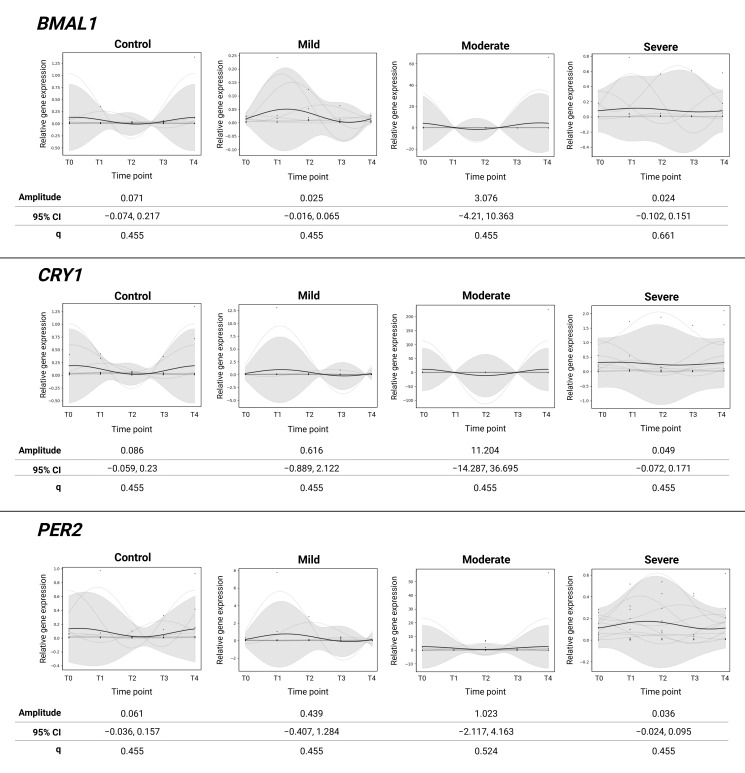
The circadian expression profiles of core clock genes (*BMAL1*, *CRY1*, and *PER2*) across OSA severity groups. Gene expression rhythms were assessed using a population-based cosinor for control, mild, moderate, and severe OSA groups. Cq values were normalized using *ACTB* and *RPL13A* as reference genes. Each subplot represents the fitted group-specific rhythm (black line), obtained by averaging individual-specific cosinor models (grey lines), and individual raw expression values at each time point (black dots). Shaded areas denote the 95% confidence interval of the fitted curve. For each panel, the amplitude estimate, its 95% confidence interval (CI), and the adjusted *p*-value (q) are reported below the plot. None of the amplitude estimates reached statistical significance after adjustment (all q > 0.05), consistent with the absence of detectable rhythmicity across severity groups. Full parameters, including mesor and acrophase, are reported in Table S10. Abbreviations: CI—confidence interval; q—adjusted *p*-value. Sampling time (*x*-axis): T0 on day 1 at 13:00, T1 on day 1 at 19:00, T2 on day 2 at 1:00, T3 on day 2 at 7:00, and T4 on day 2 at 13:00.

*BMAL1*, *CRY1*, and *PER2* Cq values were normalized with *ACTB* and *RPL13A* using the comparative Ct method [35]. Group-specific cosinor models were built by averaging individual-level fits within each OSA severity group (Figure 4, Table S10).

Group-specific cosinor analysis showed that the core clock genes *BMAL1*, *CRY1*, and *PER2* displayed detectable temporal variation across OSA severity groups, although rhythmicity did not reach statistical significance at the population level. For *BMAL1*, amplitudes were low (0.024–3.08) and mesors modest (0.026–1.43), indicating low-level, irregular expression. The acrophase, i.e., the time of peak expression, was later in mild (~20.8 h) and severe OSA (~20.5 h) groups than in controls (~13.9 h), suggesting a phase delay, though not statistically significant. For *CRY1*, amplitudes ranged from 0.05 to 11.20 (largest in the moderate OSA group) and acrophases from ~13.9 h to ~19.9 h, none significant. *PER2* amplitudes were uniformly low (0.036–1.02 in most groups), and the severe OSA group had the latest acrophase (~22.2 h), consistent with a progressive phase delay at higher disease severity, but without statistical support (Table S10).

**Figure 5:**
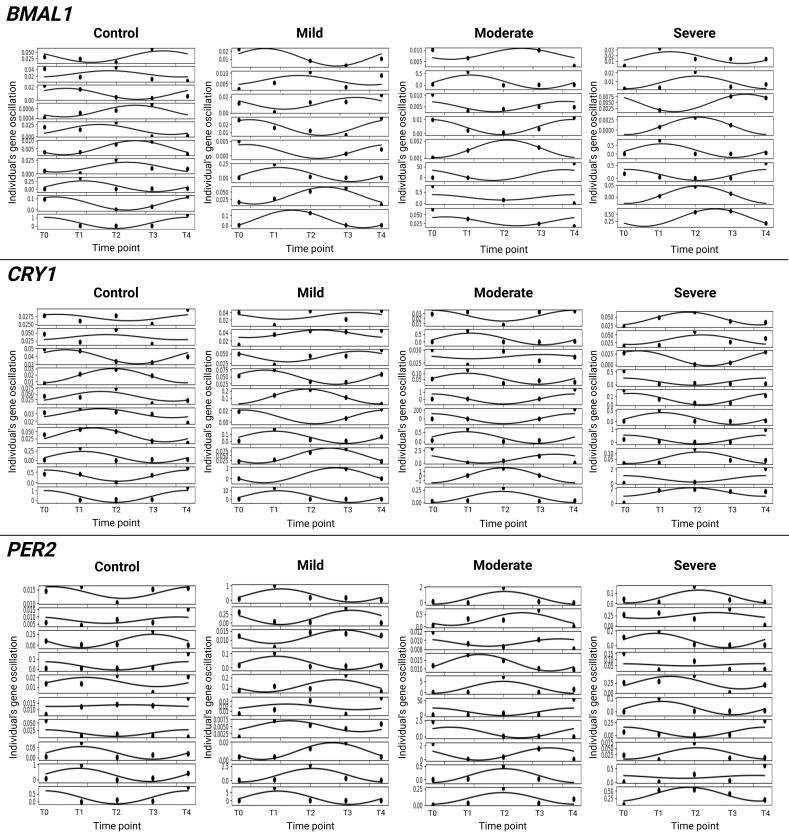
The circadian expression of core clock genes (*BMAL1*, *CRY1*, and *PER2*) of individual participants within each OSA severity group obtained from population-based cosinor analysis. Each panel represents the fitted cosinor curves (black lines) and raw expression data (black dots) for each individual participant. Rows correspond to different target genes, while columns correspond to OSA severity groups. Time points (*x*-axis) are plotted over a 24 h period, and the *y*-axis represents normalized gene expression levels. Population-based cosinor was used to estimate individual mesor, amplitude, and acrophase parameters, highlighting inter-individual variability in circadian rhythmicity within each group. This approach reveals both well-defined oscillations and flattened or phase-shifted profiles, underscoring the heterogeneity of circadian rhythms in patients with OSA. Sampling time (*x*-axis): T0 on day 1 at 13:00, T1 on day 1 at 19:00, T2 on day 2 at 1:00, T3 on day 2 at 7:00, and T4 on day 2 at 13:00.

The absence of group-level significance reflects substantial inter-individual variability rather than the universal absence of rhythmicity. Inspection of individual-level cosinor fits (Figure 5) revealed that many participants displayed clear oscillatory profiles that were obscured by group averaging, which is a known limitation of population-based chronobiology analyses in heterogeneous clinical cohorts. Detailed analysis of individual expression profiles further demonstrated substantial inter-individual variability and desynchronization of clock gene oscillations across OSA severity groups.

**Figure 6:**
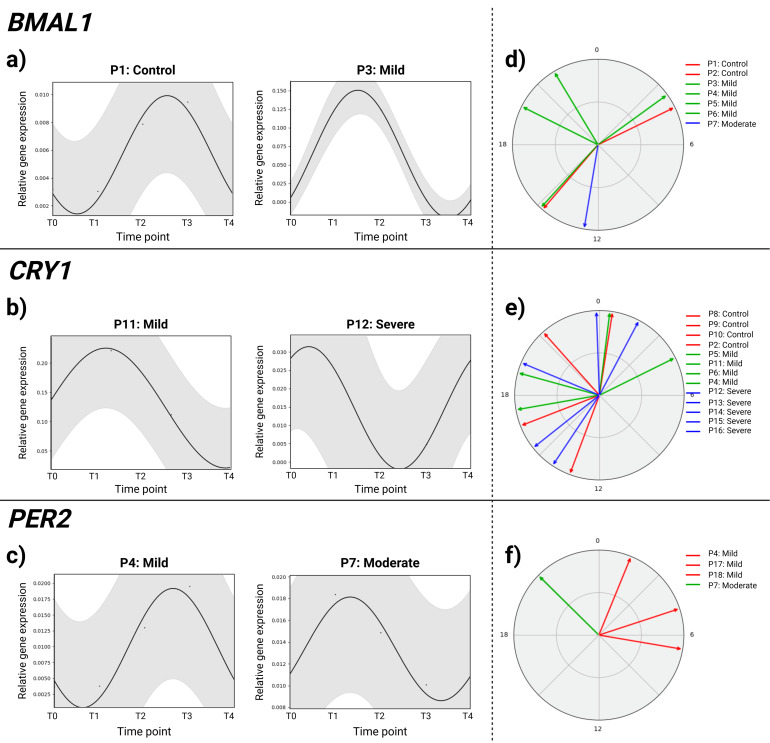
Personalized cosinor analysis of core clock genes in OSA patients and controls. Panels a–c show individual cosinor fits (black lines) with 95% confidence intervals (grey areas), and raw expression data (black dots) for representative participants, illustrating gene-specific oscillatory patterns of *BMAL1* (**a**), *CRY1* (**b**), and *PER2* (**c**). Each subplot corresponds to one participant, where “P” followed by a number denotes a specific individual (e.g., P1 = Participant 1). Examples were selected as best-case examples. Panels d–f display acrophase distributions derived from CosinorPy for *BMAL1* (**d**), *CRY1* (**e**), and *PER2* (**f**). Each line represents a different participant denoted with P and a number; different colours of the arrows apply to observed groups (red = (**d**) control or (**f**) mild, green = (**d**,**e**) mild or (**f**) moderate, blue = (**d**) moderate or (**e**) severe). Sampling time (*x*-axis): T0 on day 1 at 13:00, T1 on day 1 at 19:00, T2 on day 2 at 1:00, T3 on day 2 at 7:00, and T4 on day 2 at 13:00.

Personalized cosinor analysis (Figure 6; Tables S11 and S12) detected statistically significant rhythmicity of *BMAL1* in 7 participants, *CRY1* in 13, and *PER2* in 5, totaling 19 individuals with rhythmicity in at least one gene, largely independent of OSA severity, highlighting the highly individualized nature of circadian regulation in OSA. Acrophase distributions (Figure 6d–f) showed wide inter-individual spread with no consistent phase alignment within any OSA severity group, indicating that circadian disruption in OSA is not simply a graded function of disease severity. These findings emphasize the importance of patient-specific circadian assessment and suggest that personalized approaches may provide greater sensitivity for detecting circadian alterations in OSA.

## 4. Discussion

In this study, we addressed an important methodological challenge in circadian biology research: the identification of suitable reference genes for normalization of 24 h gene expression profiles. Accurate normalization of circadian qRT-PCR data requires reference genes that remain stable not only across disease conditions but also across the 24 h cycle, a combination that had never been validated for peripheral blood in OSA patients. Using a three-step selection framework, two independent computational tools, and progressive narrowing of an initial panel of 11 candidate reference genes, together with three additional primer sets (*CDK4*, *SDHA*, and *PPIB*), we identified *ACTB* and *RPL13A* as the most stable reference genes in human buffy coat under conditions specifically relevant to OSA: 24 h intra-patient variation, OSA severity, and the hypoxic milieu of intermittent nocturnal desaturation. This is, to our knowledge, one of the first time-resolved, blood-based reference gene validation for circadian studies in an OSA cohort.

The three-step design proved efficient. Single time-point screening (Step 1) identified and removed the most variable genes before more resource-intensive time-series work was undertaken. Extending evaluation to five time points in Step 2 revealed that *HPRT1* and *CDK4* #2, acceptable at a single time point, were in fact unstable across the circadian cycle. This underscores a general principle that reference genes for circadian studies must be validated under the same temporal conditions as the intended experiment. Final validation in 40 participants (Step 3) confirmed that *ACTB* and *RPL13A* maintained stable expression across individuals, disease severity groups, and time of day.

The stable performance of both genes is consistent with the existing literature [25,40,41]. *ACTB* has been validated as a reliable reference in blood-based circadian studies [26,29,42,43], and *RPL13A* has shown stable expression in human leukocytes under stimulation [44] and in blood under high-altitude hypoxia [45]. Particularly relevant to OSA, Wardaszka et al. (2025) demonstrated that *RPL13A* is among the most suitable reference genes in peripheral blood mononuclear cells under both normoxic and hypoxic conditions [44], which directly mirrors the intermittent hypoxia experienced by OSA patients. Together, these lines of evidence support the robustness of *RPL13A* in clinical blood-based settings characterized by disturbed oxygenation [44,45]. Conversely, both genes have shown instability in other contexts (*ACTB* in mouse lung [1] and under sleep deprivation [26]), reaffirming that reference gene suitability is tissue- and condition-specific.

Among the genes that proved unsuitable, *GAPDH* and *TBP* are particularly instructive. *GAPDH*, though widely used as a housekeeping gene [46], was eliminated in Step 1 due to high variability, and being consistent with reports that its expression is modulated by metabolic state and time of day [47,48,49]. *TBP* ranked as the least stable gene across four RefFinder algorithms in step 3 (Figure 3, Table S9), contrasting with reports of its stability in human mammary epithelial cells [18] and illustrating once more that housekeeping gene behavior cannot be assumed from the literature alone.

The case of *PPIB* illustrates a complementary pitfall, because it performed well computationally but was undetectable in 24 samples (in all triplicates) even after three repeated measurements. The number of undetected samples spread through all four groups and time points (three controls, four mild, four moderate, and three severe OSA samples). This practical failure would have been invisible if selection had relied solely on algorithm output. We therefore recommend that computational ranking always be complemented by empirical quality checks (detection rate, Cq reproducibility across all samples) before finalizing the reference gene choice. Consistent with best-practice guidance [35], using two reference genes in combination (*ACTB* and *RPL13A*) further improves normalization robustness compared to a single gene.

A key methodological strength is the integration of two independent tools. RefFinder synthesizes four established algorithms into a consensus ranking, reducing reliance on any single method’s assumptions. EndoGeneAnalyzer [38] was particularly well-suited to our multi-factor design, enabling simultaneous evaluation of stability across time of day, OSA severity, and intra-individual variation. The agreement between the two tools in selecting *ACTB* and *RPL13A* substantially strengthens confidence in the final gene pair.

Using *ACTB* and *RPL13A* for normalization, we assessed the rhythmicity of *BMAL1*, *CRY1*, and *PER2*. None showed statistically significant 24 h rhythmicity at the group level, in contrast to studies in healthy populations [5,26,28,29,42,43,50]. Several non-exclusive explanations are possible: (i) intermittent hypoxia and sleep fragmentation in OSA may dampen or desynchronize peripheral clock gene expression; (ii) the heterogeneous cellular composition of buffy coat may dilute oscillatory signals; (iii) group sizes of 10 may be insufficient to detect modest rhythmicity in a mixed clinical cohort; and (iv) five sampling points over 24 h may provide inadequate temporal resolution for low-amplitude waveforms. Comparisons with existing literature are further complicated by methodological differences across studies [5,26,28,29,42,43,50].

Despite the absence of statistical significance, non-significant phase trends deserve note. The acrophase of *BMAL1* was earlier in moderate OSA (~11.3 h) than in controls (~13.0 h), and the acrophase of *PER2* was the latest in severe OSA (~22.2 h), showing a pattern consistent with a severity-dependent phase delay in the peripheral clock. These trends, although not statistically significant, could point to a progressive disruption of the molecular circadian system with increasing disease severity. In addition, these observations reinforce the rationale for future longitudinal studies with pre- and post-treatment designs using the reference gene framework established here.

The personalized cosinor analysis revealed that circadian gene expression in OSA is highly heterogeneous, given that 19 participants displayed significant individual oscillations in at least one clock gene, yet these did not cluster by disease severity. Notably, *CRY1* exhibited the highest number of participants with significant rhythmicity (*n* = 13), followed by *BMAL1* (*n* = 7), whereas *PER2* showed the lowest number (*n* = 5). This gene-specific pattern, with some clock components more affected than others, parallels observations in OSA-related depression, where selective upregulation of specific clock genes rather than uniform clock suppression characterizes the circadian phenotype [51]. The wide spread of acrophase values within each severity group (Figure 6d–f) further suggests that individual factors (e.g., metabolic status, lifestyle, chronotype, and genetic variation) shape circadian gene expression in OSA more than disease severity alone [52]. These findings highlight the limitations of group-averaged analyses and support the use of personalized chronobiological approaches in both research and clinical chronotherapy [3,51,53,54].

OSA patients experience chronic intermittent hypoxia, sleep fragmentation, and sympathetic activation, all of which can perturb the molecular clock. Altered expression of core clock and clock-controlled genes in OSA blood is well documented [32,40,41,54], and these changes may mediate downstream comorbidities including cardiovascular disease, metabolic syndrome, and psychiatric disorders. The validated *ACTB/RPL13A* reference pair provides a reliable and practical tool for pursuing these questions in future studies.

Nevertheless, several limitations should be acknowledged. First, buffy coat is a heterogeneous mixture of cell types. Therefore, cell-type-specific circadian signals may be diluted or cancelled by this heterogeneity, and future studies using sorted leukocyte subsets or single-cell approaches may improve sensitivity. OSA may affect the proportions of different white blood cell types (e.g., the neutrophil-to-lymphocyte ratio), which could influence reference gene stability given that different leukocyte subtypes have distinct circadian rhythms [55]. However, in our cohort, neither total white blood cell counts nor differential leukocyte counts differed significantly across the four OSA severity groups (all *p* > 0.3; Supplementary Table S3). This suggests that major changes in blood cell composition are unlikely to explain the observed stability of *ACTB* and *RPL13A*. Nevertheless, small differences in cell composition cannot be completely excluded and would require sorted-cell or single-cell analyses for confirmation. Second, the initial gene screening in Step 1 relied on only two participants per OSA severity group (n = 8 total), which limits the statistical rigor of that step in isolation. However, this was a deliberate design choice to maximize screening efficiency prior to full time-series analysis, and the final reference gene selection is grounded in the expanded cohorts of Steps 2 and 3. In addition, group sizes of 10 may limit the power to detect subtle rhythmicity. Accordingly, the lack of statistically significant group-level rhythmicity for *BMAL1*, *PER2*, and *CRY1* should not be interpreted as evidence of an absent rhythm, but more likely reflects insufficient power against a background of inter-individual variability. The apparent trends observed, such as the *PER2* phase delay in severe OSA, therefore warrant confirmation in adequately powered cohorts. This interpretation must also be considered alongside several unmeasured or incompletely controlled confounders common to OSA cohorts. Individual diurnal preference (chronotype), which independently shapes clock gene phase, was assessed at enrollment using the Morningness–Eveningness Questionnaire, and participants with extreme morning or evening chronotypes were excluded. Consistent with this, chronotype distribution did not differ significantly across the four groups in the final cohort (Supplementary Table S3). While this exclusion criterion reduces the likelihood that residual chronotype differences drove the observed group-level results, it also restricts the range of diurnal preference captured in this study and may limit generalizability to individuals with more pronounced eveningness or morningness. This was a prospective clinical study in which participants were hospitalized only overnight (19:00–07:00), while daytime activities occurred in an outpatient setting under standardized instructions regarding meal timing and physical activity. Consequently, objective monitoring of light exposure and physical activity outside the hospital was not available, and their contribution to the observed phase and amplitude differences cannot be completely excluded. Similarly, BMI increased significantly with OSA severity (26.0 kg/m^2^ in controls vs. 35.6 kg/m^2^ in severe OSA; *p* = 0.005; Supplementary Table S3), consistent with the well-established comorbidity between obesity and OSA. Because adipose tissue and circulating leukocytes can each alter *BMAL1*, *CRY1*, and *PER2* expression independently of apnea severity [56], we cannot fully disentangle the effects of intermittent hypoxia from the obesity-related metabolic dysfunction in the present design. This is an inherent limitation of studying OSA in a real-world clinical cohort, where the two conditions are difficult to separate. Finally, while mean age did not differ significantly across groups (*p* = 0.416), the age range within each group remained wide (e.g., 18–64 years in the mild OSA group; Supplementary Table S3). Age-related dampening of circadian amplitude [57] may have therefore contributed to the heterogeneity observed in individual-level rhythmicity independently of OSA severity itself. Consequently, larger cohorts are needed to characterize low-amplitude oscillations and to test whether the validated reference genes remain stable in more diverse populations. We are aware that five time points at six-hour intervals may insufficiently resolve waveform dynamics with non-sinusoidal shapes or shifted phases. While a 6 h sampling interval (with an additional point at 13:00) is a logistically feasible approach for clinical cohorts, it inherently limits the statistical power of single-subject cosinor models. Consequently, the significant rhythmicity observed at the individual level in 19 out of 40 participants should be interpreted with caution, as low-density sampling schedules can elevate the risk of false-positive or false-negative rhythmic classifications. Finally, these reference genes are validated specifically for buffy coat and their stability in other blood fractions, sorted cell populations, or different tissues cannot be assumed without independent validation.

Despite these limitations, the multi-step, dual-algorithm framework presented here provides a robust, transparent, and reproducible strategy for reference gene validation. Importantly, the findings were generated in a prospective clinical circadian study, a design that is inherently demanding to conduct but provides unique insights into temporal gene expression under real-world clinical conditions. As such, the framework should be readily applicable to future clinical chronobiology studies. Furthermore, the consistent performance of *ACTB* and *RPL13A* across all conditions tested, including hypoxia, sleep fragmentation, and variation in OSA severity, supports their use as reference genes for circadian qRT-PCR studies in peripheral blood and provides a foundation for rigorous investigation of molecular clock disruption in OSA and its comorbidities.

## 5. Conclusions

*ACTB* and *RPL13A* were identified as the most stable reference genes for normalizing circadian qRT-PCR data in the buffy coat of OSA patients across a full 24 h sampling period. Their suitability was tested by personalized cosinor analysis of core clock genes (*BMAL1*, *CRY1*, *PER2*), which detected significant individual oscillations in 25 of 40 participants, consistent with inter-individual desynchrony that may obscure group-level rhythmicity. This underscores the value of personalized circadian assessment in heterogeneous clinical cohorts and the critical importance of validated, context-specific reference genes for such analyses. The reference gene pair and methodological framework established here lay the groundwork for future circadian biomarker studies in OSA, including pre- and post-treatment designs that may ultimately contribute to personalized chronotherapy in sleep medicine.

## Data Availability

The original contributions presented in this study are included in the article and supplementary material. Further inquiries can be directed to the corresponding author.

## Supplementary Materials

The following supporting information can be downloaded at: https://www.mdpi.com/article/10.3390/biom16071013/s1; Table S1: qRT-PCR primer design criteria; Figure S1: Results of step 1 candidate reference genes using EndoGeneAnalyzer; Table S2: Primer sequences, standard curve regression parameters and amplification efficiencies for all primer pairs used in this study; Table S3: Baseline demographic, chronotype, anthropometric, and hematological characteristics of the study groups, stratified by OSA severity. Figure S2: Stability evaluation of candidate reference genes using RefFinder based on Step 1 data; Table S4: Results of standard deviation and stability from EndoGeneAnalyzer, comparing different OSA severity levels from step 1 of reference gene selection; Figure S3: Evaluation of step 2 reference gene performance under different conditions using EndoGeneAnalyzer; Table S5: Results of step 1 obtained from RefFinder representing stability values of different algorithms and standard deviation from the BestKeeper algorithm; Figure S4: Comparative assessment of reference gene stability using RefFinder based on data from Step 2; Table S6: Results of standard deviations and stability from EndoGeneAnalyzer analysis of the step 2; Table S7: Results of step 2 obtained from RefFinder representing stability values of different algorithms and standard deviation from BestKeeper algorithm; Table S8: Results of standard deviations and stabilities from EndoGeneAnalyzer analysis of the step 3; Table S9: Results of step 3 obtained from RefFinder representing stability values of different algorithms and standard deviation from BestKeeper algorithm; Table S10: The rhythmicity parameters obtained on group-specific cosinor models for each group of participants and for each of the observed genes; Table S11: Individual-level cosinor parameters (amplitude, acrophase) and model fit statistics (SSE, BIC, AIC) obtained from personalized cosinor models for each participant and each observed gene; Table S12: Number of patients with statistically significant rhythmicity of core clock genes across OSA severity groups.

## Abbreviations

The following abbreviations are used in this manuscript:

AHI	Apnea-hypopnea index
BMI	Body mass index
Cq	Quantification cycle
E	Efficiency
FDR	False discovery rate
NE	Normalized expression
NF	Normalization factor
OSA	Obstructive sleep apnea
SCN	Suprachiasmatic nucleus

## Appendix A

### RNA Isolation Protocol

Total RNA was extracted using TRI Reagent LS (Sigma-Aldrich) in a 1:1 ratio to the volume of thrombocyte-leukocyte layer samples. The samples were stored at −80 °C overnight. The next day, phase separation was done following the manufacturer’s protocol. RNA precipitation was done by adding ice-cold isopropanol in a 1:1 ratio to the volume of the transferred aqueous phase. After washing the samples with 75% ethanol solution, we stored the samples at −80 °C overnight. The following day, we washed RNA two more times with a 75% ethanol solution. The RNA solubilization was performed by drying the samples for approximately 20 min at room temperature and adding an appropriate volume (app. 20 µL) of RNA-free water to the RNA pellet. Then the samples were incubated for 5 min at 55 °C.
