# Supplementary material for: Reference Genes for Circadian Profiling of Core Clock Genes in the Blood of Obstructive Sleep Apnea Patients
Source: Biomolecules. 2026 Jul 10;16(7):1013. doi: 10.3390/biom16071013 (PMC13406326; doi:10.3390/biom16071013)
Supplement: Supplementary file 1 [file biomolecules-16-01013-s001.zip › biomolecules-4391029-supplementary-update.pdf]

Supplements

Table S1: qRT-PCR primer design criteria.

Criteria type	Requirement
**PCR product size:**	from 70 to 200 bp
**Number of primers to return:**	10
**Tm:**	min = 57 °C, max = 63 °C, opt. = 60 °C
**Exon junction span:**	must span an exon-exon junction
**Organism:**	9606 (human)
**Tm difference between forward and reverse sequence:**	max 3 °C
**GC percentage:**	40-60%
The lowest value of self-complementary.	
The lowest number of potentially unintended templates.	

Table S2: Primer sequences, standard curve regression parameters and amplification efficiencies for all primer pairs used in this study.

Symbol	Gene name	NCBI Gene ID	Primer sequence (5′ – 3′)	Amplicon length (bp)	R^2	Slope range	Amplification efficiency (%)
ACTB	Actin, beta	60	FW: ACAGAGCCTCGCCTTTGCC<br>RV: GATATCATCATCCATGGTGAGCTGG	70	0.9995	-1.428	101
BMAL1	Basic helix-loop-helix ARNT like 1	406	FW: GCTCAGGAGAACCCAGGTTATC<br>RV: GCATCTGCTTCCAAGAGGCTCA	161	0.9794	-1.511	94
CDK4	Cyclin dependent kinase 4	1019	FW: GTGTATGGGGCCGTAGGAAC<br>RV: GATCAAGGGAGACCCTCACG	89	0.9975	-1.536	92
***CDK4* #2**	Cyclin dependent kinase 4, second primer sequence	1019	FW: CCATCAGCACAGTTCGTGAGGT<br>RV: TCAGTTCGGGATGTGGCACAGA	103	0.9803	-1.564	90
CRY1	Cryptochrome circadian regulator 1	1407	FW: GCAGTTGCTTGCTTCCTGACAC<br>RV: GACAGCCACATCCAACTTCCAG	125	0.9763	-1.576	88
GAPDH	Glyceraldehyde-3-phosphate dehydrogenase	2597	FW: TGGAAGGACTCATGACCACA<br>RV: TTCCCGTTCAGCTCAGGGAT	169	0.9994	-1.443	100
HPRT1	Hypoxanthine phosphoribosyl-transferase 1	3251	FW: TGCTTTCCTTGGTCAGGCAG<br>RV: TTCAAATCCAACAAAGTCTGGC	110	0.9974	-1.404	104
PER2	Period circadian regulator 2	8864	FW: AGCTGCTTGGACAGCGTCATCA<br>RV: CCTTCCGCTTATCACTGGACCT	118	0.9847	-1.490	96
PPIA	Peptidylprolyl isomerase A	5478	FW: GCCAAGACTGAGTGGTTGGAT<br>RV: GGCCTCCACAATATTCATGCC	75	0.9993	-1.460	98
PPIB	Peptidylprolyl isomerase B	5479	FW: TCTCCGAACGCAACATGAAG<br>RV: AATTCGTAGGTCAAAATACACCTTG	146	0.9950	-1.498	95
***PPIB* #2**	Peptidylprolyl isomerase B, second primer sequence	5479	FW: AACGCAGGCAAAGACACCAACG<br>RV: TCTGTCTTGGTGCTCTCCACCT	140	0.9995	-1.558	90
RPL13A	Ribosomal protein L13a	23521	FW: CGCCCTACGACAAGAAAAAGC<br>RV: TACTTCCAGCCAACCTCGTG	118	0.9994	-1.479	97
SDHA	Succinate dehydrogenase complex flavoprotein subunit A	6389	FW: GTGCATTTGGTGGACAGAGC<br>RV: TCATATCGCAGAGACCTTCCA	124	0.9996	-1.365	108
***SDHA* #2**	Succinate dehydrogenase complex flavoprotein subunit A, second primer sequence	6389	FW: GAGATGTGGTGTCTCGGTCCAT<br>RV: GCTGTCTCTGAAATGCCAGGCA	142	0.9996	-1.559	90

Symbol	Gene name	NCBI Gene ID	Primer sequence (5′ – 3′)	Amplicon length (bp)	R^2	Slope range	Amplification efficiency (%)
TBP	TATA box-binding protein	6908	FW: TGTATCCACAGTGAATCTTGGTTG<br>RV: GGTTCGTGGCTCTCTTATCCTC	124	0.9984	-1.410	103
TUBB2A	Tubulin beta 2A class IIa	7280	FW: TTGGGAGGTCATCAGCGATGAG<br>RV: AGGCTCCAGATCCACCAGGATG	151	0.9939	-1.476	98
UBC	Ubiquitin C	7316	FW: CCGGGATTTGGGTCGCAG<br>RV: TCACGAAGATCTGCATTGTCAAG	70	0.9947	-1.446	100

*Abbreviations: NCBI – National Center for Biotechnology Information; FW – forward sequence; RV – reverse sequence.

Table S3: Baseline demographic, chronotype, anthropometric, and hematological characteristics of the study groups, stratified by OSA severity.

Characteristic	Control	Mild	Moderate	Severe	p-value
**n**	10	10	10	10	—
**Sex [M/F]**	4/6	7/3	7/3	6/4	0.475
**Age [years]***	46.8 ± 11.9	46.0 ± 13.3	48.7 ± 10.2	53.9 ± 7.3	0.416
**Age range [years]**	32–65	18–64	32–60	44–64	—
**Chronotype, n [M/I/E]****	0/7/4	1/4/5	1/7/2	3/4/2	0.276
**BMI [kg/m²]***	26.0 ± 3.5	28.0 ± 3.3	30.2 ± 6.1	35.6 ± 8.7	**0.005**
**WBC [×10⁹/L]***	7.39 ± 2.12	6.99 ± 1.09	6.53 ± 1.55	7.97 ± 1.88	0.323
**Neutrophils [×10⁹/L]***	4.29 ± 1.83	3.72 ± 0.80	3.52 ± 1.19	4.65 ± 1.72	0.314
**Lymphocytes [×10⁹/L]***	2.21 ± 0.52	2.32 ± 0.56	2.22 ± 0.68	2.45 ± 0.50	0.772
**NLR***	2.00 ± 0.93	1.72 ± 0.71	1.72 ± 0.73	1.97 ± 0.87	0.784
**Monocytes [×10⁹/L]***	0.62 ± 0.19	0.70 ± 0.24	0.56 ± 0.14	0.61 ± 0.15	0.438
**Eosinophils [×10⁹/L]***	0.19 ± 0.13	0.18 ± 0.08	0.18 ± 0.07	0.20 ± 0.07	0.953
**Basophils [×10⁹/L]***	0.04 ± 0.03	0.05 ± 0.02	0.04 ± 0.03	0.04 ± 0.02	0.653

*Age, BMI, values of total and differential WBC, and NLR are presented as mean ± SD. p-values were calculated using one-way ANOVA for continuous variables and χ^2 test for categorical variables (sex, chronotype). Bold p-values indicate statistical significance ($p < 0.05$).

**Chronotype (M/I/E) was assessed using the Morningness–Eveningness Questionnaire (MEQ) and categorized as Morning, Intermediate, or Evening type.

Abbreviations: BMI, body mass index; WBC, white blood cell count; NLR, neutrophil-to-lymphocyte ratio.

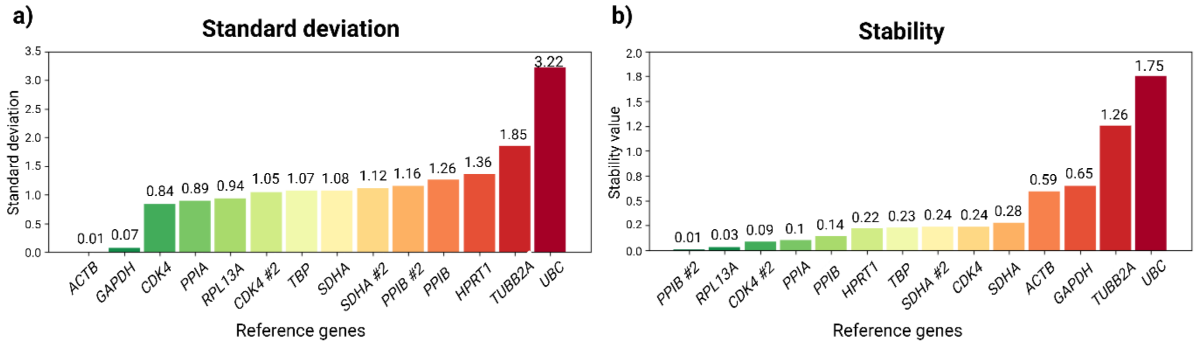

Figure S1: Results of step 1 candidate reference genes using EndoGeneAnalyzer. Reference gene stability was assessed based on (a) standard deviation and (b) stability value across all samples. Lower values indicate higher expression stability. Genes are ranked from most (left side) to least (right side) stable. The "#2" label denotes an alternative primer sequence.

Table S4: Results of standard deviation and stability from EndoGeneAnalyzer, comparing different OSA severity levels from step 1 of reference gene selection.

Gene	Standard deviation	Stability value
ACTB	0.01	0.59
GAPDH	0.07	0.65
TBP	1.07	0.23
TUBB2A	1.85	1.26
SDHA	1.08	0.28
***SDHA* #2**	1.12	0.24
PPIB	1.26	0.14
***PPIB* #2**	1.16	0.01
CDK4	0.84	0.24
***CDK4* #2**	1.05	0.09
RPL13A	0.94	0.03
HPRT1	1.36	0.22
PPIA	0.89	0.10
UBC	3.22	1.75

*Abbreviation: alternative primer sequences are marked "#2".

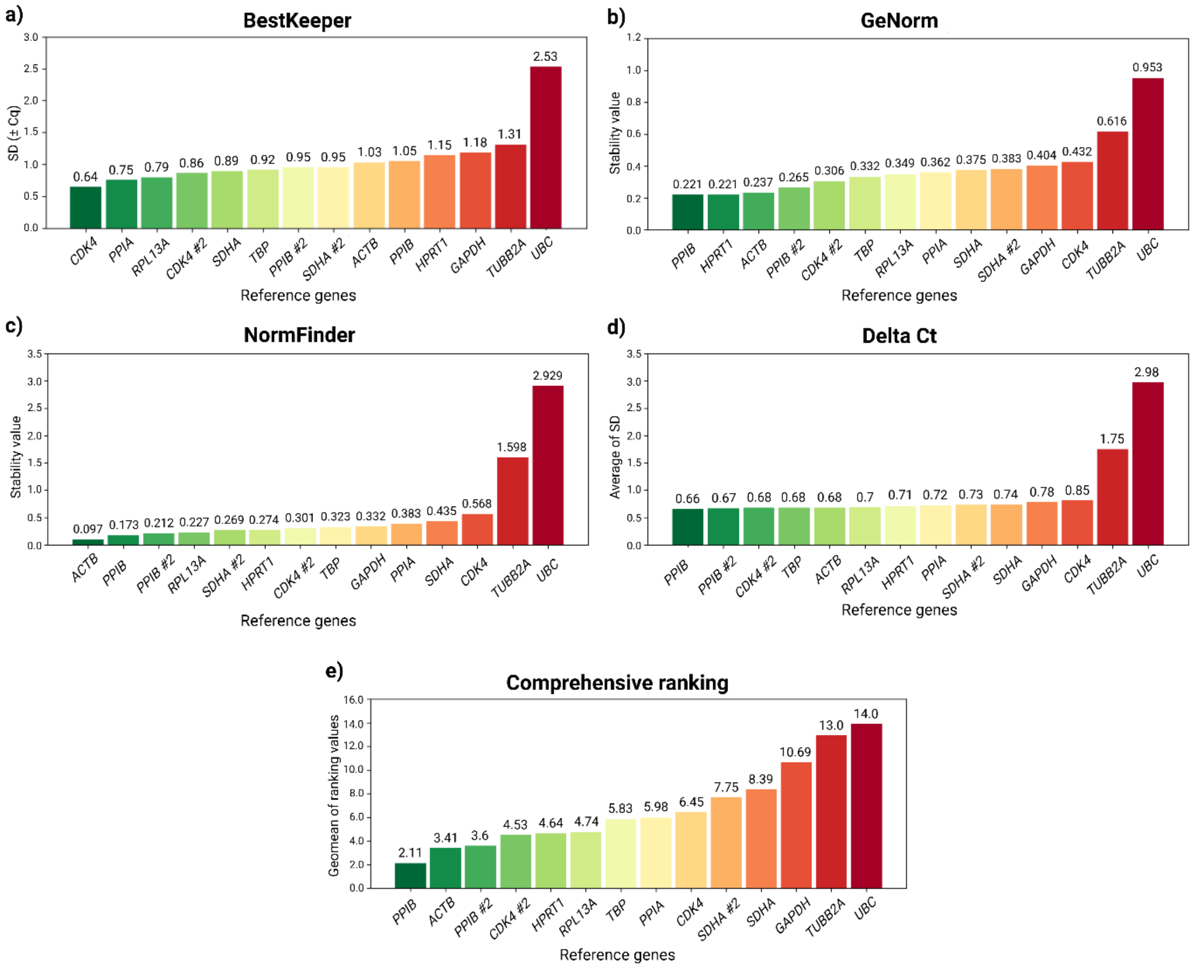

Figure S2: Stability evaluation of candidate reference genes using RefFinder based on Step 1 data. RefFinder integrates multiple statistical algorithms to assess the stability of reference genes. Panels show (a) standard deviation (SD ± Cq) values calculated by BestKeeper, (b) stability values from GeNorm, (c) NormFinder, and (d) the Delta Ct method. Lower values indicate greater gene expression stability across samples. (e) A comprehensive ranking summarizes results from all four algorithms, where lower stability values indicate higher overall gene stability. The “#2” label indicates an alternative primer sequence.

Table S5: Results of step 1 obtained from RefFinder representing stability values of different algorithms and standard deviation from the BestKeeper algorithm.

Rank	Delta Ct		BestKeeper		NormFinder		GeNorm		Comprehensive ranking	
	**Gene**	**Average of SD**	**Gene**	**SD (± Cq)**	**Gene**	**Stability value**	**Gene**	**Stability value**	**Gene**	**Geomean of ranking values**
1	*PPIB*	0.66	*CDK4*	0.64	*ACTB*	0.097	*PPIB*	0.221	*PPIB*	2.11
2	*PPIB #2*	0.67	*PPIA*	0.75	*PPIB*	0.173	*HPRT1*	0.221	*ACTB*	3.41
3	*CDK4 #2*	0.68	*RPL13A*	0.79	*PPIB #2*	0.212	*ACTB*	0.237	*PPIB #2*	3.60
4	*TBP*	0.68	*CDK4 #2*	0.86	*RPL13A*	0.227	*PPIB #2*	0.265	*CDK4 #2*	4.53
5	*ACTB*	0.68	*SDHA*	0.89	*SDHA #2*	0.269	*CDK4 #2*	0.306	*HPRT1*	4.64
6	*RPL13A*	0.70	*TBP*	0.92	*HPRT1*	0.274	*TBP*	0.332	*RPL13A*	4.74
7	*HPRT1*	0.71	*PPIB #2*	0.95	*CDK4 #2*	0.301	*RPL13A*	0.349	*TBP*	5.83
8	*PPIA*	0.72	*SDHA #2*	0.95	*TBP*	0.323	*PPIA*	0.362	*PPIA*	5.98
9	*SDHA #2*	0.73	*ACTB*	1.03	*GAPDH*	0.332	*SDHA*	0.375	*CDK4*	6.45
10	*SDHA*	0.74	*PPIB*	1.05	*PPIA*	0.383	*SDHA #2*	0.383	*SDHA #2*	7.75
11	*GAPDH*	0.78	*HPRT1*	1.15	*SDHA*	0.435	*GAPDH*	0.404	*SDHA*	8.39
12	*CDK4*	0.85	*GAPDH*	1.18	*CDK4*	0.568	*CDK4*	0.432	*GAPDH*	10.69
13	*TUBB2A*	1.76	*TUBB2A*	1.31	*TUBB2A*	1.598	*TUBB2A*	0.616	*TUBB2A*	13.00
14	*UBC*	2.98	*UBC*	2.53	*UBC*	2.929	*UBC*	0.953	*UBC*	14.00

*Abbreviations: SD – standard deviation; Cq – cycle threshold; Alternative primer sequences are marked "#2".

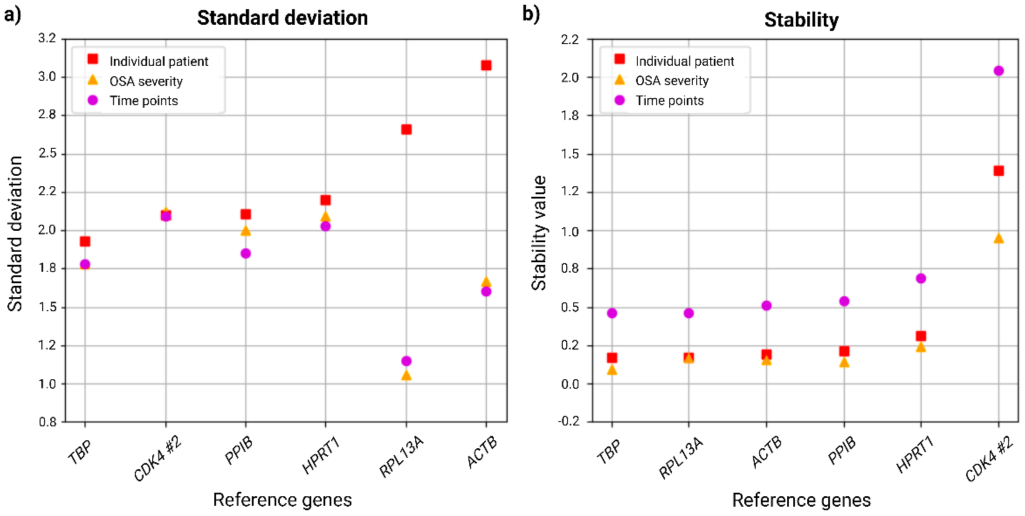

Figure S3: Evaluation of step 2 reference gene performance under different conditions using EndoGeneAnalyzer. (a) Standard deviations and (b) stability values of candidate reference genes were assessed across three conditions: individual patients (red square), OSA severity groups (orange triangles), and time points (purple circles). Lower values indicate greater expression stability. "#2" indicates an alternative primer sequence.

Table S6: Results of standard deviations and stability from EndoGeneAnalyzer analysis of the step 2.

Gene	Individual patient		OSA severity		Time points	
	SD	Stability value	SD	Stability value	SD	Stability value
PPIB	2.11	0.21	2.00	0.14	1.85	0.54
TBP	1.93	0.17	1.78	0.09	1.78	0.46
ACTB	3.08	0.19	1.67	0.16	1.60	0.51
RPL13A	2.66	0.17	1.06	0.14	1.15	0.46
CDK4 #2	2.10	1.39	2.12	0.95	2.09	2.04
HPRT1	2.20	0.31	2.09	0.24	2.03	0.69

*Abbreviations: SD – standard deviation; Alternative primer sequences are marked "#2".

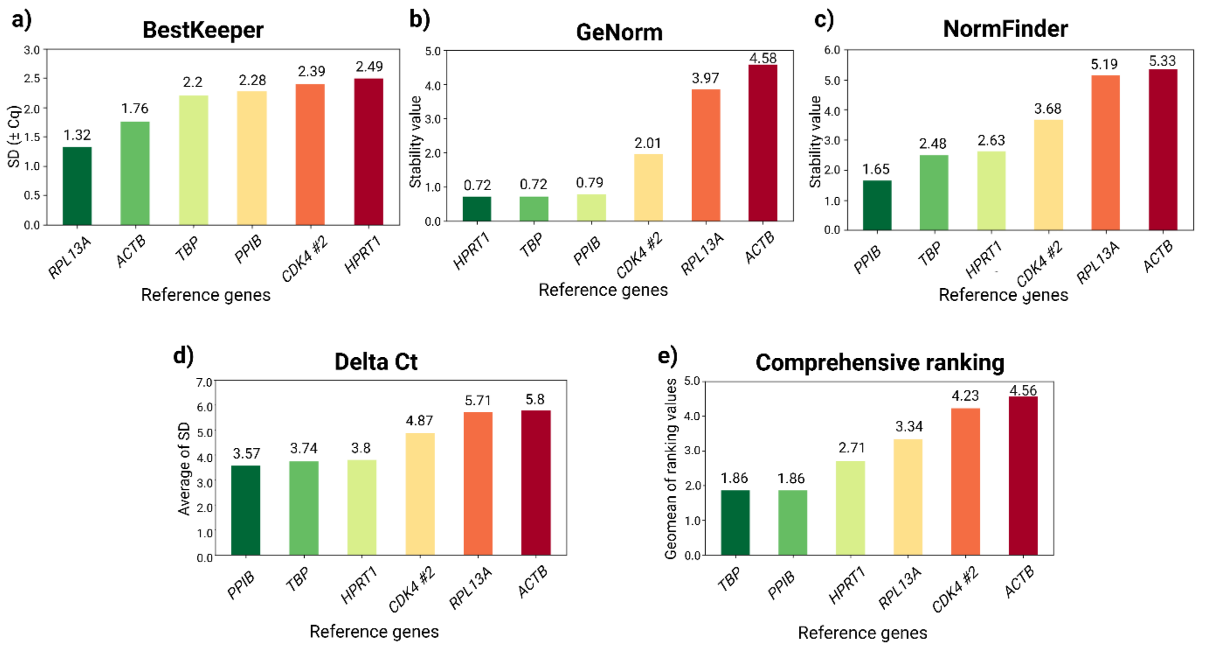

Figure S4: Comparative assessment of reference gene stability using RefFinder based on data from Step 2. The stability of candidate reference genes was evaluated using four different algorithms: (a) standard deviation calculated by BestKeeper, (b) stability ranking by GeNorm, (c) NormFinder, (d) Delta Ct method, and (e) comprehensive ranking which integrates results from all four methods. Lower values (left side) indicate greater gene expression stability. “#2” indicates an alternative primer sequence.

Table S7: Results of step 2 obtained from RefFinder representing stability values of different algorithms and standard deviation from BestKeeper algorithm.

Rank	Delta Ct		BestKeeper		NormFinder		GeNorm		Comprehensive ranking	
	**Gene**	**Average of SD**	**Gene**	**SD (± Cq)**	**Gene**	**Stability value**	**Gene**	**Stability value**	**Gene**	**Geomean of ranking values**
1	*PPIB*	3.57	*RPL13A*	1.32	*PPIB*	1.654	*HPRT1/TBP*	0.723	*TBP/PPIB*	1.86
2	*TBP*	3.74	*ACTB*	1.76	*TBP*	2.476				
3	*HPRT1*	3.80	*TBP*	2.20	*HPRT1*	2.633	*PPIB*	0.785	*HPRT1*	2.71
4	*CDK4 #2*	4.87	*PPIB*	2.28	*CDK4 #2*	3.678	*CDK4 #2*	2.012	*RPL13A*	3.34
5	*RPL13A*	5.71	*CDK4 #2*	2.39	*RPL13A*	5.192	*RPL13A*	3.974	*CDK4 #2*	4.23
6	*ACTB*	5.80	*HPRT1*	2.49	*ACTB*	5.332	*ACTB*	4.582	*ACTB*	4.56

*Abbreviations: SD – standard deviation; Cq – cycle threshold; Alternative primer sequences are marked "#2".

Table S8: Results of standard deviations and stabilities from EndoGeneAnalyzer analysis of the step 3.

Gene	Individual patient		OSA severity		Time points	
	**SD**	**Stability value**	**SD**	**Stability value**	**SD**	**Stability value**
PPIB	3.57	1.01	3.37	0.50	3.24	0.36
TBP	3.50	1.18	2.60	0.47	2.56	0.39
ACTB	2.86	1.25	2.49	0.44	2.36	0.44
RPL13A	3.03	0.95	2.53	0.23	2.49	0.24

*Abbreviation: SD – standard deviation.

Table S9: Results of step 3 obtained from RefFinder representing stability values of different algorithms and standard deviation from BestKeeper algorithm.

Rank	Delta Ct		BestKeeper		NormFinder		GeNorm		Comprehensive ranking	
	**Gene**	**Average of SD**	**Gene**	**SD (± Cq)**	**Gene**	**Stability value**	**Gene**	**Stability value**	**Gene**	**Geomean of ranking values**
1	*PPIB*	2.815	*ACTB*	1.755	*PPIB*	1.709	*PPIB*	2.514	*PPIB*	1.414
2	*ACTB*	2.886	*RPL13A*	2.184	*ACTB*	1.855	*RPL13A*	2.514	*ACTB*	1.661
3	*RPL13A*	2.972	*TBP*	2.318	*RPL13A*	2.115	*ACTB*	2.685	*RPL13A*	2.06
4	*TBP*	3.303	*PPIB*	2.715	*TBP*	2.710	*TBP*	2.994	*TBP*	3.722

*Abbreviations: SD – standard deviation; Cq – cycle threshold.

Table S10: The rhythmicity parameters obtained on group-specific cosinor models for each group of participants and for each of the observed genes.

Gene	Group	Amplitude				Mesor				Acrophase				
		**Value**	**p**	**q**	**CI**	**Value**	**p**	**q**	**CI**	**Value**	**p**	**q**	**CI**	**[h]**
BMAL1	Control	0.071	0.2988	0.4555	(-0.075, 0.217)	0.060	0.1162	0.1550	(-0.018, 0.139)	-3.642	1	1	N/A	13:55
	Mild	0.024	0.1920	0.4555	(-0.016, 0.065)	0.026	**0.0189**	0.1138	(0.006, 0.046)	-5.456	1	1	N/A	20:50
	Moderate	3.076	0.3513	0.4555	(-4.21, 10.363)	1.433	0.3304	0.3304	(-1.808, 4.674)	-2.952	1	1	N/A	11:17
	Severe	0.024	0.6613	0.6613	(-0.102, 0.151)	0.089	0.0983	0.1474	(-0.022, 0.200)	-5.376	1	1	N/A	20:32
CRY1	Control	0.085	0.2136	0.4555	(-0.059, 0.23)	0.102	**0.0391**	0.1474	(0.006, 0.198)	-3.648	1	1	N/A	13:56
	Mild	0.616	0.3785	0.4555	(-0.889, 2.122)	0.358	0.2357	0.2571	(-0.279, 0.996)	-5.208	1	1	N/A	19:54
	Moderate	11.203	0.3461	0.4555	(-14.287, 36.695)	0.160	0.0694	0.1474	(-0.016, 0.336)	-3.404	1	1	N/A	13:00
	Severe	0.049	0.3796	0.4555	(-0.072, 0.172)	0.276	0.0904	0.1474	(-0.053, 0.607)	-4.259	1	1	N/A	16:16
PER2	Control	0.060	0.1909	0.4555	(-0.036, 0.157)	0.079	0.0554	0.1474	(-0.002, 0.159)	-3.903	1	1	N/A	14:55
	Mild	0.438	0.2710	0.4555	(-0.407, 1.284)	0.324	0.0968	0.1474	(-0.072, 0.72)	-5.422	1	1	N/A	20:43
	Moderate	1.023	0.4799	0.5236	(-2.117, 4.163)	1.480	0.1590	0.1908	(-0.701, 3.663)	-3.358	1	1	N/A	12:50
	Severe	0.036	0.2107	0.4555	(-0.024, 0.095)	0.137	**0.0067**	0.0809	(0.049, 0.227)	-5.809	1	1	N/A	22:12

*Abbreviations: CI – 95% confidence interval; N/A – not applicable; p – non-adjusted p-value; q – adjusted p-value. Bold p-values indicate statistical significance ($p < 0.05$). Bold q-values indicate statistical significance after global FDR correction ($q < 0.05$).

Table S11: Individual-level cosinor parameters (amplitude, acrophase) and model fit statistics (SSE, BIC, AIC) obtained from personalized cosinor models for each participant and each observed gene.

Gene	ID	Group	Amplitude				Acrophase					SSE	BIC	AIC
			**Value**	**p**	**q**	**CI**	**Value**	**p**	**q**	**CI**	**[h]**			
BMAL1	P1	Control	0.004	**4.67E-11**	**1.189E-09**	[0.003, 0.006]	-1.116	**5.581E-14**	**1.139E-12**	[-1.41, -0.825]	04:16	0	-54.89	-53.72
	P2	Control	0.060	**7.47E-06**	**7.617E-05**	[0.034, 0.087]	2.435	**5.969E-11**	**8.697E-10**	[1.705, 3.164]	14:42	0.0004	-21.44	-19.59
	P3	Mild	0.086	**0**	**0**	[0.083, 0.089]	0.541	**0**	**0**	[0.513, 0.569]	21:56	0	-42.44	-40.59
	P4	Mild	0.029	**0.0001**	**0.001**	[0.014, 0.044]	-0.944	**0.0004**	**0.0020**	[-1.468, -0.420]	03:36	0.0003	-30.03	-28.86
	P5	Mild	0.009	**0.0110**	0.070	[0.002, 0.017]	2.404	**2.889E-08**	**2.679E-07**	[1.555, 3.253]	14:49	0.0001	-36.74	-35.58
	P6	Mild	0.123	**0.0381**	0.176	[0.007, 0.240]	1.106	**0.0075**	**0.0182**	[0.295, 1.916]	19:47	0.0144	-10.25	-9.08
	P7	Moderate	0.005	**0.0003**	**0.002**	[0.002, 0.008]	2.978	**8.546E-20**	**2.179E-18**	[2.337, 3.618]	12:38	0	-45.58	-44.41
	P8	Control	0.000	0.1508	0.275	[-0.0, 0.0003]	-1.111	0.111	0.182	[-2.4751, 0.254]	04:14	0	-62.23	-60.39
	P9	Control	0.008	0.1224	0.270	[-0.002, 0.019]	1.940	**0.002**	**0.006**	[0.734, 3.146]	16:35	0.0001	-33.82	-32.65
	P10	Control	0.009	0.1088	0.252	[-0.002, 0.02]	-0.758	0.267	0.395	[-2.098, 0.581]	02:53	0.0002	-32.69	-31.52
	P13	Severe	0.010	0.1773	0.304	[-0.005, 0.025]	0.841	0.210	0.315	[-0.474, 2.157]	20:47	0.0003	-30.49	-29.32
	P16	Severe	0.391	0.0518	0.185	[-0.003, 0.786]	1.192	**0.006**	**0.016**	[0.336, 2.048]	19:26	0.1626	1.89	3.06
	P18	Mild	0.007	0.1819	0.304	[-0.003, 0.018]	-2.272	**0.001**	**0.003**	[-3.592, -0.952]	08:40	0.0001	-34.03	-32.86
	P20	Control	0.022	0.1935	0.309	[-0.011, 0.056]	-2.327	**0.003**	**0.008**	[-3.835, -0.820]	08:53	0.0006	-19.75	-17.91
	P21	Control	0.009	0.5215	0.598	[-0.019, 0.037]	0.040	0.982	0.982	[-3.470, 3.550]	23:50	0.0011	-23.08	-21.90
	P22	Moderate	0.002	0.5801	0.630	[-0.006, 0.011]	-1.069	0.560	0.697	[-4.664, 2.526]	04:05	0	-32.49	-30.65
	P23	Moderate	0.286	0.0537	0.185	[-0.005, 0.577]	1.199	**0.006**	**0.016**	[0.336, 2.061]	19:25	0.0884	-1.16	0.01
	P24	Severe	0.007	0.1156	0.262	[-0.002, 0.016]	-0.326	0.664	0.744	[-1.798, 1.146]	01:14	0.0001	-34.45	-33.28
	P25	Mild	0.011	0.2500	0.336	[-0.008, 0.030]	1.720	**0.008**	**0.019**	[0.448, 2.992]	17:25	0.0001	-27.71	-25.87
	P26	Mild	0.002	0.4068	0.506	[-0.003, 0.007]	0.236	0.858	0.912	[-2.352, 2.823]	23:06	0	-40.19	-39.02
	P27	Moderate	0.002	0.2392	0.336	[-0.002, 0.006]	-2.999	**0.002**	**0.006**	[-4.869, -1.129]	11:27	0	-43.34	-42.17
	P29	Control	0.011	0.2183	0.323	[-0.006, 0.027]	0.466	0.573	0.705	[-1.157, 2.089]	22:13	0.0003	-28.86	-27.69
	P36	Control	0.172	0.0839	0.225	[-0.023, 0.3672]	1.172	**0.017**	**0.037**	[0.207, 2.136]	19:31	0.0398	-5.14	-3.97
	P37	Severe	0.213	0.1347	0.270	[-0.066, 0.4916]	2.770	**0.000**	**0.002**	[1.226, 4.314]	13:25	0.1128	0.06	1.23
	P39	Moderate	0.013	0.6951	0.737	[-0.0503, 0.0755]	2.300	0.303	0.433	[-2.074, 6.673]	15:12	0.0012	-16.89	-15.06
	P40	Control	0.671	0.1635	0.288	[-0.2731, 1.6157]	2.906	**0.000**	**0.000**	[1.499, 4.313]	12:53	0.4644	6.89	8.74
CRY1	P1	Control	0.006	0.2496	0.336	[-0.004, 0.0152]	0.421	0.639	0.735	[-1.339, 2.182]	22:23	0.0001	-34.35	-33.18
	P2	Control	0.263	**0.0122**	0.070	[0.057, 0.468]	2.783	**3.449E-09**	**4.398E-08**	[1.859, 3.706]	13:22	0.0614	-2.98	-1.81
	P3	Mild	6.606	0.0528	0.185	[-0.0812, 13.2924]	1.153	**0.009**	**0.020**	[0.291, 2.016]	19:35	46.8809	30.21	31.38
	P4	Mild	0.040	**2.28E-07**	**3.329E-06**	[0.025, 0.055]	-1.110	**4.334E-09**	**4.911E-08**	[-1.482, -0.739]	04:15	0.0003	-30.03	-28.86
	P5	Mild	0.025	**5.05E-07**	**6.436E-06**	[0.015, 0.035]	1.743	**1.674E-22**	**5.691E-21**	[1.393, 2.094]	17:20	0.0001	-34.79	-33.61

Gene	ID	Group	Amplitude				Acrophase					SSE	BIC	AIC
			Value	p	q	CI	Value	p	q	CI	[h]			
	P6	Mild	0.087	**0.0370**	0.176	[0.005, 0.168]	1.302	**0.0013**	**0.0049**	[0.507, 2.097]	19:02	0.0069	-13.89	-12.73
	P7	Moderate	0.048	0.1452	0.270	[-0.0164, 0.1116]	1.359	**0.019**	**0.039**	[0.221, 2.497]	18:48	0.0043	-16.31	-15.14
	P8	Control	0.011	**3.93E-09**	**8.025E-08**	[0.007, 0.015]	-0.156	0.3572	0.4991	[-0.489, 0.177]	00:36	0	-37.47	-35.63
	P9	Control	0.009	**0.0088**	0.060	[0.002, 0.015]	1.940	**9.309E-08**	**7.913E-07**	[1.228, 2.653]	16:35	0	-38.56	-37.39
	P10	Control	0.025	**0.0157**	0.084	[0.005, 0.045]	0.727	0.0604	0.1082	[-0.032, 1.487]	21:13	0.0005	-27.43	-26.26
	P11	Mild	0.102	**4.66E-125**	**2.375E-123**	[0.094, 0.111]	-0.124	**0.0030**	**0.0094**	[-0.207, -0.042]	00:29	0	-30.85	-29.01
	P12	Severe	0.017	**7.59E-63**	**2.579E-61**	[0.015, 0.019]	2.242	**1.129E-165**	**5.759E-164**	[2.082, 2.403]	15:26	0	-43.33	-41.49
	P13	Severe	0.018	**1.66E-05**	**0.00015**	[0.009, 0.026]	0.035	0.8937	0.9302	[-0.487, 0.559]	23:52	0.0001	-35.31	-34.13
	P14	Severe	0.066	**0.0124**	0.070	[0.014, 0.118]	2.554	**2.343E-08**	**2.389E-07**	[1.658, 3.451]	14:15	0.0038	-16.94	-15.77
	P15	Severe	0.050	**0.0451**	0.185	[0.001, 0.099]	-0.487	0.4014	0.5532	[-1.625, 0.651]	01:52	0.0035	-17.34	-16.17
	P16	Severe	0.273	**0.0473**	0.185	[0.003, 0.542]	1.175	**0.0061**	**0.0164**	[0.336, 2.016]	19:30	0.076	-1.91	-0.74
	P18	Mild	0.014	0.1313	0.270	[-0.004, 0.031]	-2.408	**0.000**	**0.001**	[-3.621, -1.194]	09:11	0.0004	-28.73	-27.55
	P19	Severe	0.560	0.2442	0.336	[-0.382, 1.502]	-0.139	0.890	0.930	[-2.120, 1.842]	00:31	1.2868	12.2313	13.40
	P20	Control	0.001	0.4837	0.567	[-0.002, 0.004]	2.327	0.131	0.208	[-0.689, 5.344]	15:06	0	-46.42	-45.25
CRY1	P21	Control	0.008	0.7552	0.778	[-0.042, 0.058]	0.145	0.964	0.974	[-6.141, 6.430]	23:26	0.0013	-16.67	-14.83
	P22	Moderate	0.011	0.1051	0.252	[-0.002, 0.024]	2.875	**0.000**	**0.001**	[1.444, 4.306]	13:01	0.0002	-30.56	-29.38
	P23	Moderate	0.378	0.0576	0.185	[-0.012, 0.768]	1.164	**0.009**	**0.020**	[0.285, 2.043]	19:33	0.1593	1.79	2.96
	P24	Severe	0.015	0.2229	0.325	[-0.009, 0.039]	-0.588	0.531	0.677	[-2.425, 1.249]	02:14	0.0008	-24.41	-23.24
	P25	Mild	0.009	0.5627	0.622	[-0.022, 0.041]	-2.231	0.147	0.223	[-5.242, 0.781]	08:31	0.0011	-23.14	-21.97
	P26	Mild	0.009	0.4057	0.506	[-0.012, 0.031]	-0.669	0.620	0.735	[-3.313, 1.975]	02:33	0.0006	-25.83	-24.66
	P27	Moderate	0.001	0.7259	0.756	[-0.004, 0.005]	-2.011	0.409	0.557	[-6.788, 2.765]	07:40	0	-42.57	-41.40
	P28	Severe	0.140	0.4059	0.506	[-0.190, 0.470]	2.925	**0.040**	0.075	[0.136, 5.713]	12:49	0.1587	1.77	2.94
	P29	Control	0.021	0.1939	0.309	[-0.011, 0.053]	0.402	0.616	0.735	[-1.169, 1.972]	22:27	0.0013	-22.27	-21.10
	P30	Moderate	0.679	0.4749	0.567	[-1.183, 2.539]	2.876	**0.004**	**0.011**	[0.936, 4.816]	13:00	0.9016	9.55	11.39
	P31	Moderate	112.60	0.4796	0.567	[-199.56, 424.76]	2.880	**0.004**	**0.012**	[0.919, 4.84]	13:00	25366.19	50.53	52.37
	P32	Moderate	0.307	0.2172	0.323	[-0.180, 0.794]	1.403	0.084	0.142	[-0.186, 2.991]	18:38	0.1237	1.61	3.45
	P33	Moderate	0.873	0.3682	0.482	[-1.028, 2.774]	-2.514	**0.019**	**0.039**	[-4.619, -0.409]	09:36	4.2444	18.19	19.37
	P36	Control	0.149	0.1006	0.250	[-0.029, 0.326]	1.192	**0.021**	**0.042**	[0.178, 2.205]	19:26	0.0329	-6.09	-4.92
	P37	Severe	0.334	0.2021	0.312	[-0.179, 0.846]	2.810	**0.002**	**0.008**	[0.995, 4.625]	13:16	0.382	6.16	7.33
	P39	Moderate	0.111	0.0723	0.200	[-0.01, 0.231]	-0.211	0.748	0.812	[-1.500, 1.078]	00:48	0.0212	-8.29	-7.12
	P40	Control	0.633	0.1851	0.305	[-0.303, 1.569]	2.904	**0.000**	**0.001**	[1.425, 4.383]	12:54	0.456	6.82	8.67

Gene	ID	Group	Amplitude				Acrophase					SSE	BIC	AIC
			Value	p	q	CI	Value	p	q	CI	[h]			
	P1	Control	0.001	0.8773	0.877	[-0.007, 0.009]	-0.755	0.915	0.943	[-14.674, 13.165]	02:53	0.0001	-35.81	-34.64
	P2	Control	0.430	0.0951	0.243	[-0.075, 0.935]	1.446	**0.005**	**0.013**	[0.447, 2.445]	18:28	0.267	4.37	5.54
	P3	Mild	3.933	0.0547	0.185	[-0.079, 7.946]	1.162	**0.009**	**0.020**	[0.293, 2.031]	19:33	16.871	25.09	26.27
	P4	Mild	0.009	**2.97E-08**	**5.057E-07**	[0.006, 0.013]	-1.253	**1.085E-13**	**1.844E-12**	[-1.584, -0.923]	04:47	0	-45.45	-44.27
	P5	Mild	0.046	0.0662	0.193	[-0.003, 0.096]	1.225	**0.008**	**0.019**	[0.321, 2.128]	19:19	0.0025	-18.92	-17.75
	P6	Mild	0.002	0.2452	0.336	[-0.001, 0.005]	0.663	0.420	0.564	[-0.949, 2.274]	21:28	0	-45.74	-44.56
	P7	Moderate	0.004	**6.07E-06**	**6.88E-05**	[0.003, 0.007]	0.793	**9.239E-05**	**0.0005**	[0.396, 1.191]	20:58	0	-50.33	-49.16
	P8	Control	0.041	0.3931	0.506	[-0.053, 0.134]	2.929	**0.034**	0.066	[0.216, 5.642]	12:48	0.0127	-10.84	-9.67
	P9	Control	0.156	0.0636	0.191	[-0.009, 0.320]	-1.847	**0.000**	**0.000**	[-2.739, -0.954]	07:03	0.0282	-6.87	-5.69
	P10	Control	0.011	0.5495	0.621	[-0.025, 0.047]	2.937	0.137	0.215	[-0.934, 6.808]	12:46	0.0019	-20.47	-19.29
	P11	Mild	0.071	0.2036	0.312	[-0.038, 0.179]	-1.427	**0.042**	0.078	[-2.801, -0.053]	05:26	0.0129	-10.78	-9.61
	P12	Severe	0.099	0.0581	0.185	[-0.003, 0.201]	1.448	**0.001**	**0.005**	[0.568, 2.328]	18:28	0.0109	-11.62	-10.45
	P13	Severe	0.062	0.0724	0.200	[-0.006, 0.129]	-0.479	0.460	0.602	[-1.749, 0.792]	01:49	0.0065	-14.23	-13.06
	P14	Severe	0.155	0.1061	0.252	[-0.033, 0.343]	0.819	0.147	0.223	[-0.287, 1.924]	20:52	0.0392	-5.22	-4.05
	P15	Severe	0.023	0.1795	0.304	[-0.011, 0.057]	-0.408	0.641	0.735	[-2.123, 1.307]	01:33	0.0016	-21.14	-19.97
	P16	Severe	0.049	0.0504	0.185	[-0.0001, 0.099]	1.181	**0.007**	**0.016**	[0.329, 2.032]		0.0025	-18.90	-17.73
PER2	P17	Mild	1.216	**0.0189**	0.097	[0.200, 2.232]	-0.388	0.4389	0.5815	[-1.371, 0.595]	01:29	1.4951	12.98	14.15
	P18	Mild	0.004	**0.0479**	0.185	[3.493e-05, 0.008]	-1.738	**4.990E-05**	**0.0003**	[-2.578, -0.898]	06:38	0	-44.60	-43.43
	P19	Severe	0.229	**0.0022**	**0.016**	[0.082, 0.376]	-0.022	0.9531	0.9721	[-0.768, 0.723]	00:05	0.0309	-6.42	-5.25
	P20	Control	0.002	0.1456	0.270	[-0.0007, 0.005]	2.611	**0.001**	**0.004**	[1.056, 4.167]	14:01	0	-46.19	-45.02
	P21	Control	0.002	0.5537	0.621	[-0.005, 0.009]	-2.913	0.116	0.188	[-6.546, 0.719]	11:07	0.0001	-36.99	-35.81
	P22	Moderate	0.819	0.0634	0.191	[-0.046, 1.684]	-0.334	0.600	0.728	[-1.581, 0.913]	01:16	1.0886	11.39	12.57
	P23	Moderate	0.260	0.1375	0.270	[-0.083, 0.602]	-1.091	0.100	0.167	[-2.392, 0.209]	04:10	0.1404	1.16	2.33
	P24	Severe	0.073	0.6003	0.645	[-0.201, 0.347]	-1.161	0.528	0.677	[-4.764, 2.441]	04:26	0.0877	-1.19	-0.03
	P25	Mild	0.459	0.0910	0.238	[-0.073, 0.990]	1.023	**0.046**	0.085	[0.016, 2.030]	20:05	0.3012	4.97	6.14
	P26	Mild	0.208	0.1371	0.270	[-0.066, 0.482]	-2.094	**0.000**	**0.002**	[-3.234, -0.954]	07:59	0.0796	-1.69	-0.51
	P27	Moderate	0.001	0.5030	0.583	[-0.002, 0.003]	-2.736	0.078	0.137	[-5.779, 0.307]	10:27	0	-48.29	-47.12
	P28	Severe	0.009	0.8604	0.869	[-0.096, 0.115]	3.119	0.637	0.735	[-9.819, 16.056]	12:05	0.0157	-9.79	-8.62
	P29	Control	0.005	0.4374	0.538	[-0.007, 0.017]	1.128	0.305	0.433	[-1.029, 3.285]	19:41	0.0002	-32.83	-31.66
	P30	Moderate	2.694	0.1410	0.270	[-0.893, 6.282]	-0.262	0.745	0.812	[-1.837, 1.313]	01:00	18.7583	25.63	26.80
	P31	Moderate	13.256	0.4754	0.567	[-23.149, 49.661]	2.880	0.082	0.142	[-0.369, 6.129]	12:59	1932.051	48.80	49.97
	P32	Moderate	0.838	0.5667	0.622	[-2.030, 3.707]	2.112	0.305	0.433	[-1.921, 6.145]	15:55	2.925	14.26	16.10
	P33	Moderate	0.880	0.2659	0.352	[-0.670, 2.431]	-2.300	**0.005**	**0.013**	[-3.895, -0.705]	08:47	2.6578	15.86	17.03

Gene	ID	Group	Amplitude				Acrophase					SSE	BIC	AIC
			Value	p	q	CI	Value	p	q	CI	[h]			
PER2	P34	Moderate	0.260	0.1275	0.270	[-0.074, 0.594]	-0.293	0.656	0.743	[-1.579, 0.994]	01:07	0.0581	-1.42	0.42
	P35	Mild	0.006	0.7004	0.737	[-0.023, 0.035]	0.793	0.740	0.812	[-3.883, 5.469]	20:58	0.0009	-23.86	-22.69
	P36	Control	0.031	0.1250	0.270	[-0.009, 0.071]	1.300	**0.018**	**0.038**	[0.219, 2.379]	19:02	0.0016	-21.10	-19.93
	P37	Severe	0.097	0.2047	0.312	[-0.053, 0.248]	2.857	**0.002**	**0.007**	[1.029, 4.685]	13:05	0.0329	-6.09	-4.93
	P38	Severe	0.052	0.8208	0.837	[-0.400, 0.505]	-2.911	0.548	0.690	[-12.401, 6.578]	11:07	0.2743	4.50	5.68
	P39	Moderate	0.088	0.1624	0.288	[-0.036, 0.212]	-0.240	0.777	0.834	[-1.899, 1.420]	00:54	0.0224	-8.02	-6.85
	P40	Control	0.416	0.2365	0.336	[-0.273, 1.105]	2.910	**0.001**	**0.003**	[1.255, 4.566]	12:53	0.247	4.37	6.21

*Cosinor parameters could not be estimated for some gene–patient combinations due to an insufficient number of data points. Abbreviations: CI – 95% confidence interval; p – non-adjusted p-value; q – adjusted p-value. Bold p-values indicate statistical significance ($p < 0.05$). Bold q-values indicate statistical significance after FDR correction ($q < 0.05$).

Table S12: Number of patients with statistically significant rhythmicity of core clock genes across OSA severity groups.

Gene	OSA severity	Number of patients	SUM
	Control	2	
BMAL1	Mild	4	7
	Moderate	1	
	Control	4	
CRY1	Mild	4	13
	Severe	5	
	Mild	3	
PER2	Moderate	1	5
	Severe	1	
